# Split-belt treadmill training improves gait symmetry and lower limb function in patients with stroke

**DOI:** 10.1038/s41598-025-98322-3

**Published:** 2025-05-08

**Authors:** Chunfang Wang, Qun Zhang, Siyan Hou, Dan Guo, Xuemin Han, Weiguang Huo, Ying Zhang

**Affiliations:** 1 Rehabilitation Medical Department, Tianjin Union Medical Centre, Tianjin, 300121 China; 2 Tianjin Institute of Rehabilitation, Tianjin, 300121 China; 3 The First Clinical Medical College of Nanjing Medical University, Nanjing, 211166 Jiangsu China; 4 College of Artificial Intelligence, Nankai University, Tianjin, 300350 China

**Keywords:** Stroke, Split-belt treadmill training, Gait symmetry, Lower extremity function, Physiology, Stroke

## Abstract

Split-belt treadmill training (SBTT) enhances gait symmetry in patients with stroke (PwS) by improving sensorimotor adaptation, However, it remains unclear whether repeated SBTT leads to long-term acquisition of gait adaptation skills and whether it improves functional walking in PwS. This study aimed to investigate the effect of SBTT on gait symmetry and lower limb function in PwS. We designed a parallel-group randomized controlled experiment. PwS who have the ability to stand and walk unassisted for at least 30 min were included in this study. They were randomly assigned to either rehabilitation with SBTT or with tied-belt treadmill training (TBTT). The treatment was provided once per day, five times per week for four weeks(total of 20 treatments). Gait analysis including step length, gait speed, temporal and spatial asymmetry and clinical assessment of lower limb function using Fugl-Meyer Assessment - lower extremity (LEFM), the Berg Balance Scale (BBS), the Wisconsin Gait Scale(WGS), and the Timed Up and Go Test (TUGT) were measured at baseline, 2 weeks post intervention, 4 weeks post intervention, and at follow-up after 4 weeks finishing the exercise. Two-way repeated measures ANOVA was used for intergroup comparisons between the two groups at different time points. In addition, Pearson correlation analysis was used to test the relationship between the improvement of clinical assessment scales and changes in gait parameters. Results showed that SBTT could effectively and efficiently improve the spatial asymmetry and speed of gait, as well as lower limb balance and walking function in PwS. In addition, the improvement of functional walking was positively correlated with the decrease of spatial asymmetry, and the increase of step length of paretic leg. In Conclusion, a 4-week SBTT intervention could effectively improve gait asymmetry and, consequently, enhance walking and lower limb function in PwS with independent walking ability.

Stroke, also known as cerebrovascular accident, is a sudden neurological disorder caused by impaired perfusion of the cerebral blood vessels^1^. The incidence of stroke in China is generally rising^2^. It is estimated that the incidence of cerebrovascular disease in China will increase by approximately 50% by 2030 compared to 2010^3^. Strokes can lead to various motor, sensory, cognitive, speech, and swallowing dysfunctions. Hemiparesis is the most common clinical complication in patients with stroke (PwS), and lower limb dysfunction is a significant determinant of long-term disability in PwS^4^. Approximately 80% of PwS experience difficulty in walking, and approximately 25% of survivors of stroke have residual gait impairment even after rehabilitation, which affects their quality of daily life severely^5^.

Lower limb motor dysfunction in PwS is mainly characterized by speed, endurance, and gait asymmetry^6^. The manifestation of gait asymmetry primarily encompasses the asymmetry of temporal parameters such as the stance and swing phases between the sound and affected sides, as well as the asymmetry of spatial parameters such as stride length^7^. Research indicates that spatiotemporal gait asymmetry poststroke may result in compromised dynamic balance and an elevated risk of falls. Consequently, during the rehabilitation of PwS, it is of importance to focus on the spatiotemporal asymmetry of their gait and to explore methods to ameliorate this asymmetry.

Error augmentation (EA), an emerging concept in motor learning and rehabilitation for PwS, refers to the utilization of patients’ erroneous sensory feedback to enhance their adaptation to new environments and promote the recovery of motor functions post-neurological damage^8^. Following a stroke, the neural feedback system is usually compromised, yet feedback plays an important role in motor learning during the rehabilitation process^9^. The EA training approach artificially amplifies visual and tactile feedback during movement errors, thereby facilitating the recovery of neural feedback system. Previous studies^10,11^show that emphasizing and magnifying errors during incorrect movements might stimulate motor learning in PwS.

Split-belt treadmill training (SBTT) induces controlled gait perturbation through asynchronous belt velocities, capitalizing on error-based adaptation (EA) principles to amplify proprioceptive feedback and recalibrate asymmetric movement patterns in stroke survivors^12,13^. Emerging evidence substantiates the preserved neuroplastic capacity of persons with stroke (PwS) to acquire novel locomotor strategies through this paradigm^11^. Related studies confirms SBTT’s efficacy (improving walking speed and gait symmetry) eliciting acute-phase improvements in PwS^14,15^. However, it remains unknown whether repeated split-belt walking training leads to long-term acquisition of gait adaptation skills and whether it improves functional walking in PwS.

In this study, we designed a randomized controlled experiment to compare the improvement in gait and lower limb function in PwS treated with SBTT and tied-belt treadmill training (TBTT). Furthermore, we investigated the relationship between gait improvement and lower limb function improvement, aiming to find some factors for SBTT in improving the rehabilitation of lower limb function.

## Results

### Participants characteristics

Table 1 shows the baseline characteristics of PwS. A total of 27 participants (14 SBTT and 13 TBTT) were included in this study. There was no significant differences in basic information between the two groups of PwS.

**Table 1:** Baseline characteristics of participants.

Group	*N*	Gender		Time to stroke (months)	Type of stroke		Age	Lesion side	
		Male	Female		Hemorrhage	Ischemia		Left	Right
SBTT	14	11	3	1.0(1.0, 3.0)	3	11	66(56,72)	8	6
TBTT	13	10	3	1.0(1.0, 3.0)	1	12	62(47,69)	9	4
χ^2^/Z		0.36		-0.725	1.008		-0.9	0.422	
*P*		0.58		0.583	0.315		0.375	0.516	

### Gait measurement results

**Fig. 1:**
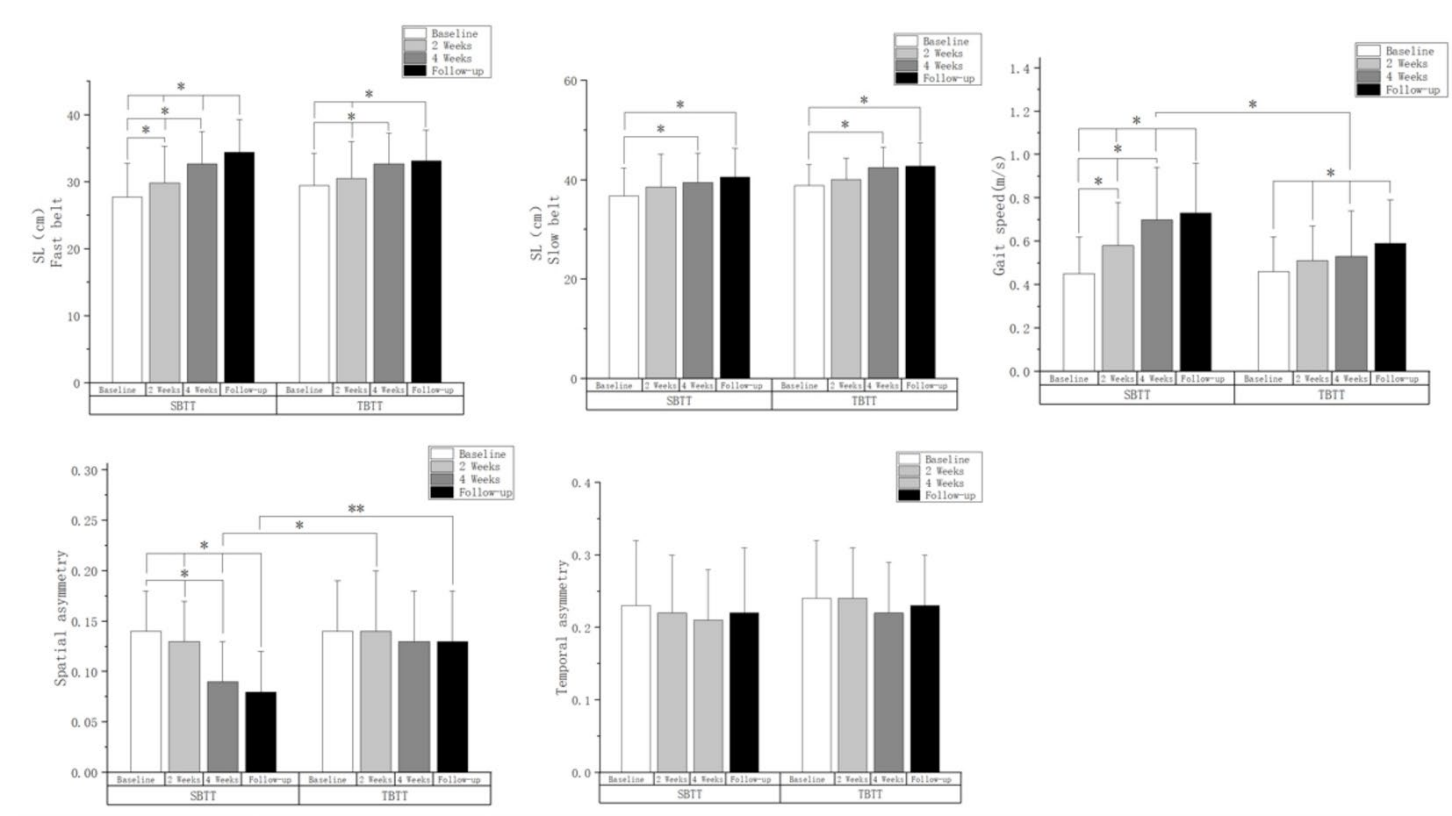
Post-hoc analysis result of gait parameters among different intervention time points.

Results of gait parameters assessed at baseline, 2 weeks post intervention, 4 weeks post intervention, and at follow-up are shown in Table 2. The analysis of variance showed significant differences in step length, spatial asymmetry, and gait speed at the four time points in the SBTT group (*P* < 0.01). But the TBTT group only demonstrated significant differences in step length and gait speed across the four time points (*P* < 0.01). Post-hoc analysis result was shown in Fig. 1. After 2 weeks of treatment, the SBTT group exhibited significant improvements in step length on the fast belt and gait speed, whereas the TBTT group’s improvements were observed only after 4 weeks of treatment. Comparative analysis between the groups indicated that, after 4 weeks of treatment, patients in the SBTT group showed significantly improved spatial asymmetry and gait speed compared to those in the TBTT group (*P* < 0.05). However, follow-up results indicated that, 4 weeks after cessation of treatment, only the spatial asymmetry index in the SBTT group remained better than that in the TBTT group.

**Table 2:** Patients’ walking features assessed at baseline, 2 weeks post intervention, 4 weeks post intervention, and at follow-up after 4 weeks finishing the exercise.

	Group	Baseline	2 Weeks	4 Weeks	Follow-up (8 weeks)	F	*P*
SL_Fast belt_ (cm)	SBTTT	27.69 ± 5.08	29.83 ± 5.49	32.68 ± 4.80	34.44 ± 4.83	33.743	0.000
	TBTTT	29.44 ± 4.79	30.51 ± 5.46	32.71 ± 4.56	33.13 ± 4.58	10.265	0.000
F		0.943	0.114	0.009	0.579		
P		0.340	0.738	0.984	0.453		
SL_Slow belt_ (cm)	SBTT	36.78 ± 5.57	38.59 ± 6.57	39.49 ± 5.83	40.56 ± 5.78	4.958	0.007
	TBTT	38.84 ± 4.26	40.02 ± 4.38	42.45 ± 4.08	42.79 ± 4.71	5.534	0.004
F		1.291	0.495	2.601	1.345		
P		0.265	0.488	0.118	0.256		
Spatial asymmetry	SBTT	0.14 ± 0.04	0.13 ± 0.04	0.09 ± 0.04	0.08 ± 0.04	8.070	0.001
	TBTT	0.14 ± 0.05	0.14 ± 0.06	0.13 ± 0.05	0.13 ± 0.05	0.282	0.838
F		0.005	0.290	5.703	10.074		
P		0.943	0.595	0.024	0.004		
Temporal asymmetry	SBTT	0.23 ± 0.09	0.22 ± 0.08	0.21 ± 0.07	0.22 ± 0.09	1.583	0.217
	TBTT	0.24 ± 0.08	0.24 ± 0.07	0.22 ± 0.07	0.23 ± 0.07	1.520	0.233
F		0.005	0.003	0.003	0.000		
P		0.945	0.958	0.956	0.988		
Gait speed(m/s)	SBTT	0.45 ± 0.17	0.58 ± 0.20	0.70 ± 0.24	0.73 ± 0.23	39.032	0.000
	TBTT	0.46 ± 0.16	0.51 ± 0.16	0.53 ± 0.21	0.59 ± 0.20	19.870	0.000
F		0.044	1.312	4.335	3.682		
P		0.836	0.262	0.047	0.065		

Note: a indicates significant *P* < 0.05 compared with before-treatment in the group; b indicates significant *P* < 0.05 compared with middle-treatment in the group; c indicates significant *P* < 0.05 compared with after-treatment in the group.

### Clinical assessment results

**Fig. 2:**
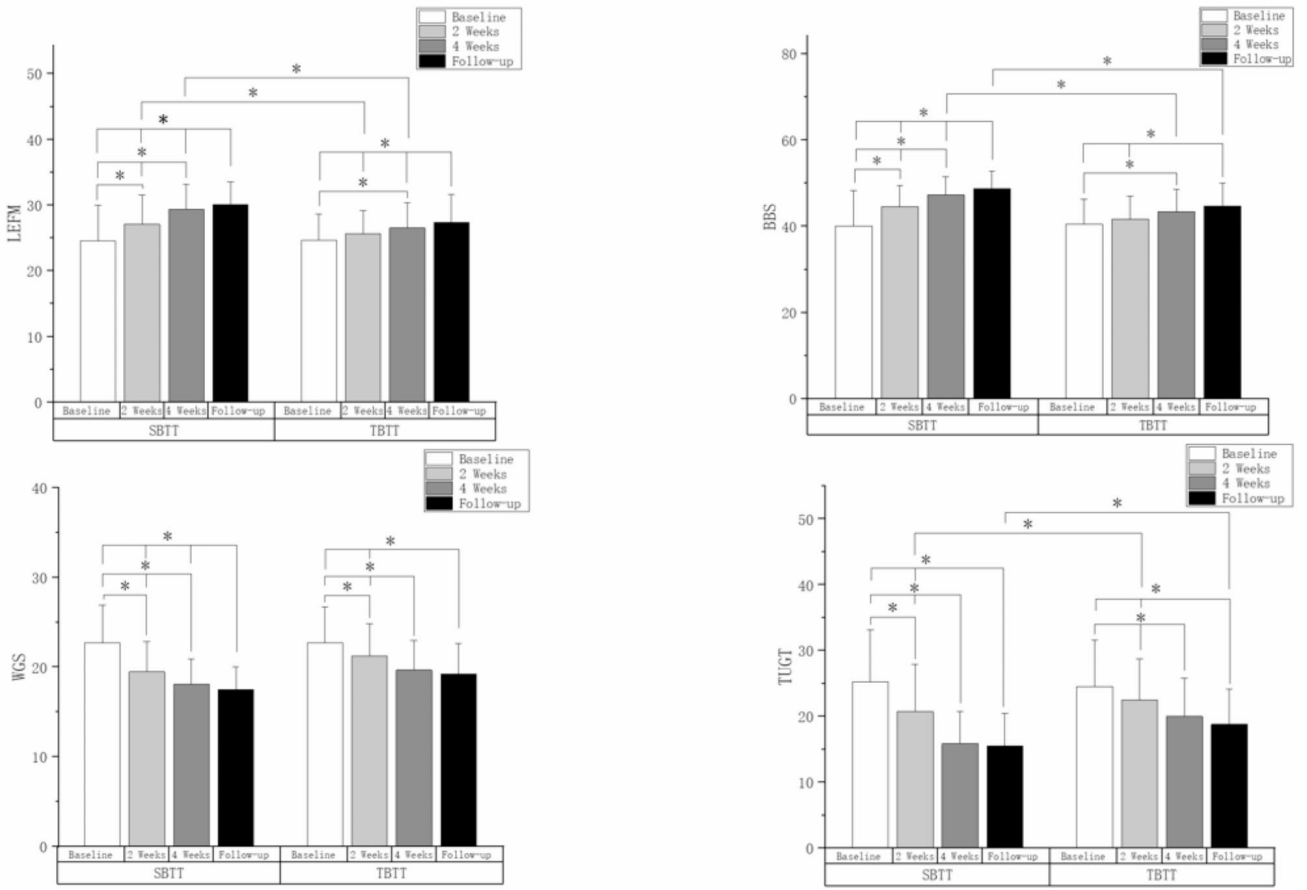
Post-hoc analysis result of lower limb clinical assessment scales among different intervention time points.

Results of clinical measurements of lower-limb function assessed at baseline, 2 weeks post intervention, 4 weeks post intervention, and at follow-up are shown in Table 3. The variance analysis showed significant differences in LEFM, BBS, WGS, and TUGT scores between the SBTT group and TBTT group at the four time points (*P* < 0.01). Post-hoc analysis result was shown in Fig. 2. Although both groups experienced significant improvements in the above-mentioned indices after 4 weeks of treatment, the SBTT group demonstrated significant improvements as early as 2 weeks into the treatment, while the TBTT group only showed improvement in WGS after 2 weeks (*P* < 0.05). The comparison between groups indicated that, after 4 weeks of treatment, the SBTT group showed more significant improvements in LEFM, BBS, and TUGT compared to the TBTT group (*P* < 0.05). Follow-up results further indicated that the improvement in BBS in the SBTT patients remained significantly better than that in the TBTT group even 4 weeks after the cessation of treatment.

**Table 3:** PwS’ clinical measurements of lower-limb function assessed at baseline, 2 weeks post intervention, 4 weeks post intervention, and at follow-up.

	Group	Baseline	2 Weeks	4 Weeks	Follow-up (8 weeks)	F	*P*
LEFM	SBTT	24.53 ± 5.42	27.07 ± 4.45	29.40 ± 3.76	30.07 ± 3.43	32.002	0.000
	TBTT	24.60 ± 4.05	25.67 ± 3.46	26.53 ± 3.80	27.33 ± 4.27	7.245	0.001
F		0.001	0.926	4.322	3.733		
P		0.970	0.344	0.047	0.064		
BBS	SBTT	40.07 ± 8.30	44.53 ± 4.88	47.33 ± 4.17	48.67 ± 4.06	17.716	0.000
	TBTT	40.47 ± 5.85	41.73 ± 5.32	43.47 ± 5.07	44.67 ± 5.33	8.992	0.000
F		0.023	2.253	5.206	5.345		
P		0.880	0.145	0.030	0.028		
WGS	SBTT	22.73 ± 4.20	19.47 ± 3.38	18.07 ± 2.81	17.47 ± 2.56	24.539	0.000
	TBTT	22.73 ± 3.99	21.20 ± 3.65	19.67 ± 3.29	19.20 ± 3.43	8.637	0.000
F		0.177	1.823	2.050	2.463		
P		0.678	0.188	0.163	0.128		
TUGT	SBTT	25.20 ± 7.97	20.73 ± 7.18	15.87 ± 4.90	15.47 ± 4.96	25.382	0.000
	TBTT	24.53 ± 7.03	22.47 ± 6.29	20.00 ± 5.82	18.80 ± 5.37	9.407	0.000
F		0.059	0.495	4.431	3.119		
P		0.810	0.488	0.044	0.088		

Note: a indicates significant *P* < 0.05 compared with baseline; b indicates significant *P* < 0.05 compared with the 2 weeks post intervention; c indicates significant *P* < 0.05 compared with the 4 weeks post intervention.

### The relationship between the improvement of clinical assessment scales and changes in gait parameters

**Fig. 3:**
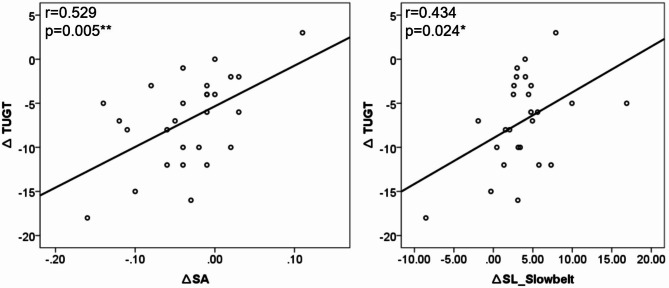
Relationships between the improvement of TUGT scales and changes in spatial asymmetry (SA) and in step length of slow belt (SL_Slowbelt).

Results of the pearson correlation analysis showed that the decrease of spatial asymmetry positively correlated to the improvement of TUGT significantly (*P* < 0.01) and the increase of step length of slow belt negatively correlated to the improvement of TUGT significantly (*P* < 0.05). No significant correlation was observed between the improvement of other clinical assessment and changes of gait parameters. Figure 3 shows the results with significant correlation.

## Discussion

The main finding of the present study is that compared to TBTT, SBTT could accelerate the changes in step length of fast belt and gait speed, and improve the spatial asymmetry of gait and gait speed better. As for lower limb function, SBTT could increase the rehabilitation process of lower limbs for PwS, and improve the functions of lower limbs, balance, and functional walking more effectively. In addition, this study found that the improvement of spatial symmetry in PwS has a positive effect on the rehabilitation of functional walking, while the increase in step length of the paretic leg has a negative effect on the improvement of walking function.

Sensorimotor adaptability serves as the foundation for motor function recovery in PwS. Rehabilitation treatment based on the principles of EA demonstrate significant effects in enhancing sensorimotor adaptation poststroke. Neurophysiological studies suggest that erroneous sensory predictions may facilitate human sensorimotor adaptation^16^. Molier et al.^10^ found that when PwS exhibit incorrect movement patterns, emphasizing and amplifying these errors can stimulate their sensorimotor adaptability.

The asymmetric gait pattern, a clinically significant feature in persons with stroke (PwS), serves as a critical determinant of locomotor instability, substantially increasing fall risk and compromising ambulatory efficiency^17^. Despite conventional rehabilitation efforts, persistent deficits in both temporal (e.g., stance phase duration) and spatial (e.g., step length) symmetry parameters frequently endure post-intervention^18^. Our results show that SBTT could improve the spatial asymmetry and gait speed efficiently and effectively. The therapeutic protocol strategically positioned the paretic limb (characterized by prolonged step length) on the slow belt and the non-paretic limb (shorter step length) on the fast belt. During the initial adaptation phase, the slower belt enforced prolonged paretic limb stance durations, while the accelerated non-paretic belt constrained step length. Through iterative error-based adaptation (EA), participants progressively recalibrated stride dynamics via cerebellar-mediated error correction: elongating strides on the fast belt while shortening those on the slow belt^12^. Crucially, these adapted patterns persisted during a four-weeks training, maintaining spatial asymmetry and speed improvements.

This three-stage process - deliberate asymmetry amplification, sensorimotor recalibration, and persistent aftereffect retention - mechanistically explains EA-driven symmetry enhancement. Our findings align with evidence confirming that improvement in SL was primarily achieved by increasing the length of the short step^19^, and PwS preserves interlimb adaptation capacity with the transfer efficiency to overground walking^20,21^. Recent study reports advantage of SBTT over TBTT in improving step length symmetry with single session^22^. These reports are consistent with our findings, but lacking the long-term effects including the relationship between train duration and symmetry retention, time-dependent decay of rehabilitation effects post-intervention. Our study provides novel clinical evidence on the effects of a 4-week SBTT intervention on gait symmetry and lower limb functional outcomes, including the sustained retention of therapeutic gains for 4 weeks post-intervention.

Our study did not observe SBTT’s improvement in temporal symmetry indices. A possible explanation is that stance time, being a reactive parameter, heavily relies on peripheral feedback from hip flexor afferents, limb load receptors, and cutaneous feedback^23^. Consequently, temporal parameters like stance phase duration are highly sensitive to speed differences on the split-belt treadmill, allowing immediate changes through feedback mechanisms but not adapting during SBTT or being retained as an aftereffect. Previous research also showed reduced step length asymmetry but unchanged stance time asymmetry in PwS after multiple SBTT sessions^24^, aligning with our results.

The perturbations induced by SBTT largely elicit automatic responses in the lower limbs of PwS, requiring minimal additional attention, similar to sudden changes in walking direction or sharp turns in daily life^25^. Our results indicate that SBTT can efficiently and effectively improve the TUGT scores of PwS. Betschart et al.^26^ found that after 2–3 weeks of SBTT, PwS’ walking speed got improved, and this improvement persisted for at least one month. This is consistent with our findings. Furthermore, SBTT’s efficacy in enhancing walking speed has been confirmed in patients with neurological disorders such as cerebellar ataxia and Parkinson’s disease^27,28^.

While cross-sectional studies have failed to uncover a significant correlation between gait symmetry and walking velocity^29,30^, longitudinal studies have revealed that post-stroke changes in gait symmetry negatively impact gait efficiencies and postural control^31^. Our study found that the improvement in TUGT positively correlated with the reduction in spatial asymmetry and negatively correlated with the increase in the step length of the paretic leg, indirectly suggesting that SBTT enhances walking efficiency by improving gait symmetry.

Gama et al.^32^ conducted a six-week treadmill walking training program, three times a week, for 14 chronic PwS. Their results showed improved scores on the LEFM scale, consistent with our findings. We speculate that the two belts of the split-belt treadmill can regulate the bilateral lower limbs at different speeds. Repeated exposure to bilateral uncoupled walking conditions may better activate the paralyzed muscles, optimizing the timing, amplitude, and coordination of muscle activity, thereby promoting the rehabilitation of lower limb gait and balance functions. Previous studies have also affirmed SBTT’s positive effects on balance function in PwS^27,33^.

In Conclusion, a 4-week SBTT intervention could effectively improve gait asymmetry and, consequently, enhance walking and lower limb function in PwS with independent walking ability.

This study has certain limitations. Firstly, this study is restricted by limited sample and single-center study. Multi-center trails with larger sample size helps to achieve more reliable clinical results. Moreover, we only included PwS who could stand and walk unassisted for at least 30 min at a speed of 1.5 m/s. Thus, our conclusions may only be applicable to mild lower limb dysfunction stroke populations rather than the entire population. In future research, we consider conducting SBTT trials on PwS with severe lower limb dysfunction with the assistance of exoskeletons^34^ to assess the clinical rehabilitation effects of SBTT on this population. Additionally, we used limited gait parameters in this study. Future research will incorporate more detailed gait parameters such as the asymmetry of stance phase and swing phase to explore the reasons behind the improvement in lower limb gait and motor function achieved through SBTT treatment.

## Methods

### Participants

**Fig. 4:**
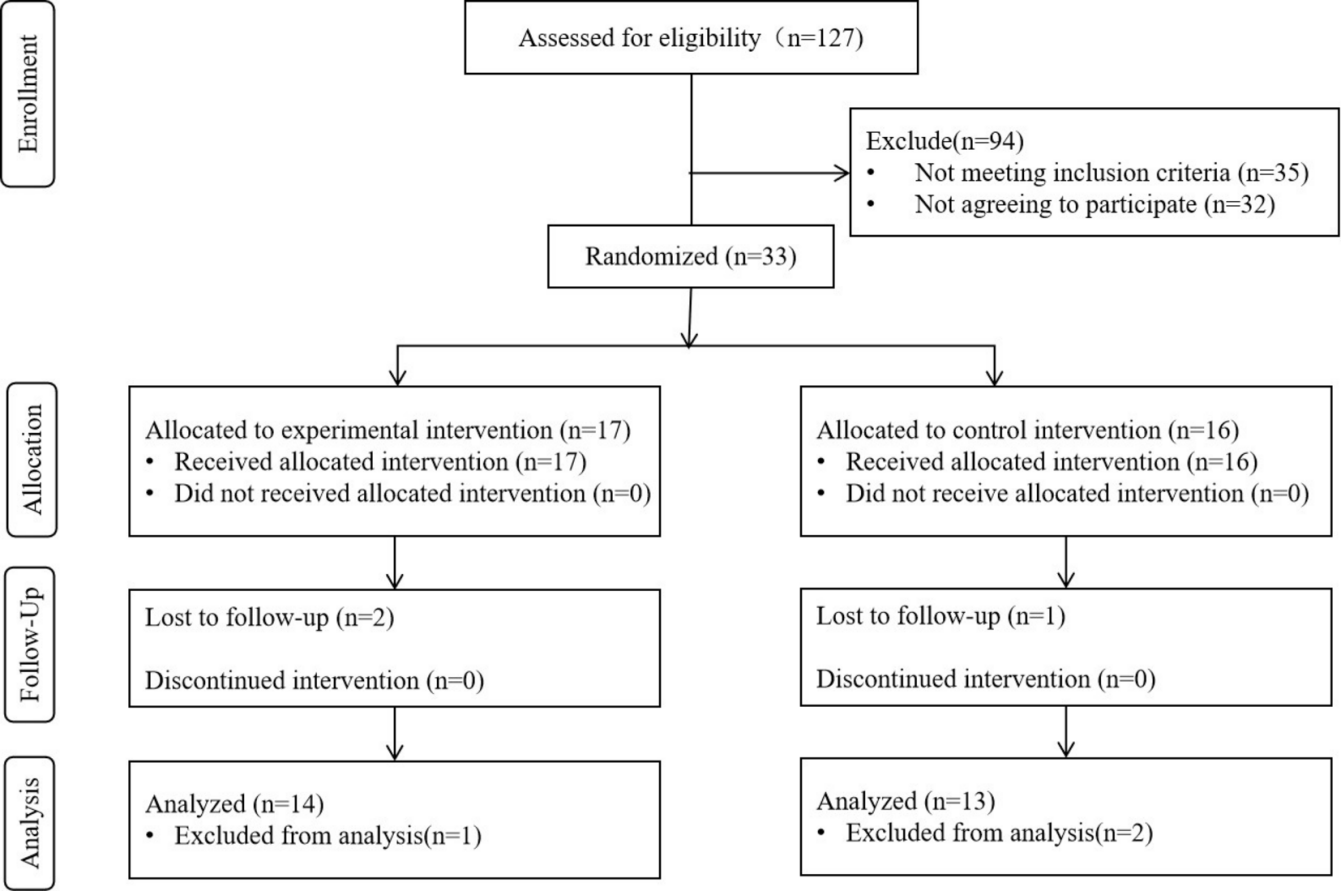
Consort flow diagram of the study.

This is a parallel-group randomized controlled experiment. A total of twenty seven patients with post-stroke lower limb dysfunction who were hospitalized at Tianjin Union Medical Centre between February 2023 and September 2023 were included in this study. Participants were randomly assigned to either rehabilitation with split-belt treadmill training (SBTT) (the experimental group) or with tied-belt treadmill training (TBTT) (the control group). A consort flow diagrams is shown in Fig. 4. The inclusion criteria are as follows: ① 25–75 years old, male or female; ② Patients with unilateral cortical ischemic stroke or hemorrhagic stroke diagnosed by Magnetic Resonance Imaging(MRI)according to the diagnostic criteria for stroke formulated by the 4th National Academic Conference on Cerebrovascular Disease; ③ Ability to stand and walk unassisted for at least 30 min with the speed of 1.5 m/s; ④ No surgical treatment; ⑤ No neurological or musculoskeletal disorders that inhibit gait due to causes other than stroke; ⑥ Sign the informed consent form for the experiment. The exclusion criteria are as follows: ①Patients with severe cognitive impairment (Montreal Cognitive Assessment score less than 25); ②Ataxia due to cerebellar stroke; ③ Subjects who take drugs that affect the nervous system; ④ Subjects who have received botulinum toxin treatment.

This study was approved by the Ethics Committee of Tianjin Union Medical Centre (2023C10) and have been performed in accordance with the Declaration of Helsinki. All methods were performed in accordance with the relevant guidelines and regulations. All participants or their guardians signed an informed consent form before enrollment.

### Procedures

**Fig. 5:**
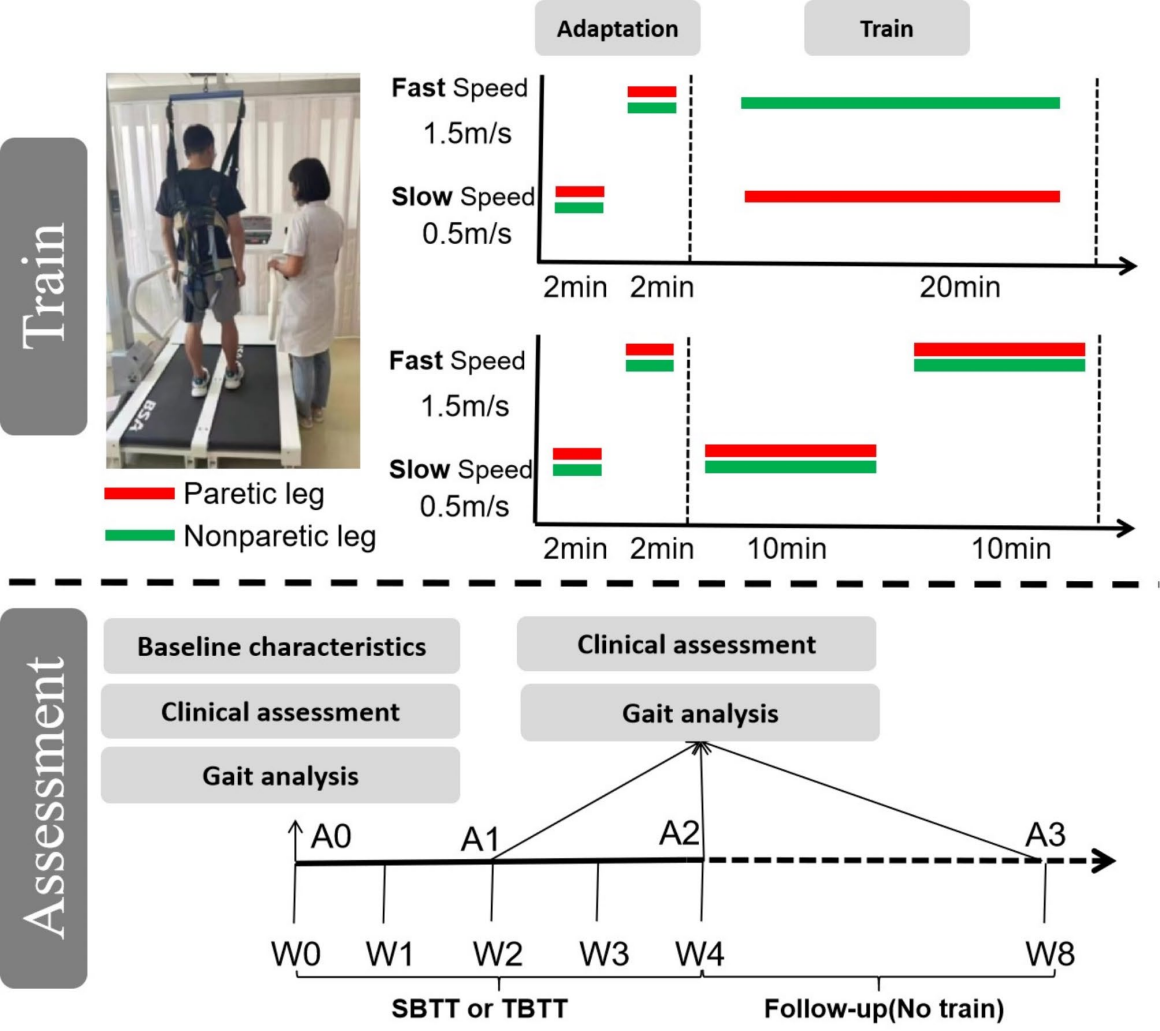
The experimental paradigm of assessment and train.

Participants were randomly allocated to either the experimental group receiving split-belt treadmill training (SBTT) or the control group receiving tied-belt treadmill training (TBTT) using a computer-generated block randomization sequence (block size = 4) stratified by baseline Fugl-Meyer Assessment scores. The intervention protocol comprised 20 sessions administered once daily, five times weekly over four consecutive weeks. Outcome assessments including gait analysis and clinical scales were conducted at four time points: baseline (A0), mid-intervention (A1: 2 weeks post intervention), post-intervention (A2: 4 weeks post intervention), and follow-up (A3: 8 weeks from baseline). A schematic overview of the experimental timeline is presented in Fig. 5.

### Split-belt treadmill training (SBTT) and tied-belt treadmill training (TBTT) protocol

The custom-designed dual-belt treadmill (Sunnyou Inc., Henan Province, China; Model DT-2022) features two independently controlled belts with an adjustable speed range of 0.05–2.8 m/s and safety harness system. Both groups underwent standardized 24-minute sessions consisting of: ①Adaptation phase (4 min): 0–2 min: Bilateral slow-speed walking (slow speed: 0.5 m/s), and 2–4 min: Bilateral fast-speed walking (fast speed: 1.5 m/s); ②Training phase (20 min): SBTT group: Asymmetric walking with paretic limb on slow belt (0.5 m/s) and non-paretic limb on fast belt (1.5 m/s), and TBTT group: Symmetric walking with bilateral slow-speed (0.5 m/s; 0–10 min) followed by bilateral fast-speed (1.5 m/s; 10–20 min). The speed alteration of the two sessions was set in the pre-treatment equipment setup. PwS will receive verbal instructions from the treadmill operator prior to each velocity change.

### Gait analysis

Overground gait characteristics were assessed through 10-meter walk tests using bilateral inertial measurement units (LPMS-B2, micro wireless transmission attitude sensor, Guangzhou, China; 400 Hz sampling rate; bluetooth transmission) affixed to the dorsal surface of footwear at the metatarsophalangeal joint. The step speed, step length (SL) of the paretic and nonparetic sides, spatial asymmetry (SA) and temporal asymmetry (TA) rates were calculated using MATLAB. In order to calculate step length and support phase time, the gait phase should be accurately recognized by using Inertial Measurement Units placed on both feet, i.e., detecting heel contact and toe-off moments. The gait phase detection algorithm was proposed in our previous work^35^. The support phase time was calculated as the time period from the moment of heel contact to the next moment of toe-off. The step length was calculated as follows:

$$\begin{aligned} s &= \sqrt {{s_x}^{2}+{s_y}^{2}} \hfill \\ {s_x} &= \iint {{a_{Gx}}(t)d}tdt \hfill \\ {s_y} &= \iint {{a_{Gy}}(t)d}tdt \hfill \\ \end{aligned}$$

where s denotes the step length, while sx and sy are the components of the XY-direction of the displacement in the global reference frame G. Let the XY plane of G be parallel to the treadmill. aGx and aGy are the accelerations of the IMUs in the XY-axis direction in G, respectively. t is the sampling step during the swing phase. The SA and TA were calculated as follows:

$${\text{Spatial}}\;{\text{Asymmetry}} = \left( {{\text{step}}\;{\text{length}}_{{{\text{slow}}\;{\text{belt}}}} - {\text{step}}\;{\text{length}}_{{{\text{fast}}\;{\text{belt}}}} } \right)/({\text{step}}\;{\text{length}}_{{{\text{slow}}\;{\text{belt}}}} + {\text{step}}\;{\text{length}}_{{{\text{fast}}\;{\text{belt}}}});$$

$$\begin{aligned}{\text{Temporal}}\;{\text{Asymmetry}} &= ({\text{support}}\;{\text{phase}}\;{\text{time}}_{{{\text{slow}}\;{\text{belt}}}}\\ &\quad - {\text{support}}\;{\text{phase}}\;{\text{time}}_{{{\text{fast}}\;{\text{belt}}}} )/({\text{support}}\;{\text{phase}}\;{\text{time}}_{{{\text{slow}}\;{\text{belt}}}} + {\text{support}}\;{\text{phase}}\;{\text{time}}_{{{\text{fast}}\;{\text{belt}}}}).\end{aligned}$$

### Clinical assessment of lower-limb function

Standardized outcome measures were administered by blinded assessors. All participants underwent a clinical assessment of lower-limb function and walking ability using Fugl-Meyer Assessment - lower extremity (LEFM), the Berg Balance Scale (BBS), the Wisconsin Gait Scale(WGS), and the Timed Up and Go Test (TUGT). The Fugl-Meyer Assessment - lower extremity (LEFM) was used to assess the PwS’ lower-extremity motor function, which had a total of 34 points, with higher scores indicating better function^36^. The Berg balance scale (BBS) was used to assess the balance function of the patients. The BBS scale consists of 14 entries, with 4 points for each entry and a total score of 0–56 points, with a higher total score indicating a better balance function^37^. The “Timed up and Go Test” (TUGT) is a rapid quantitative assessment of functional walking ability that measures the time required for a patient to stand up from a standard armchair, walk a distance of 3 m, turn around, walk back, and sit down again. The participants can practice once or twice before the test to familiarize with the entire testing process. A testing time of less than 10 min indicates walking ease (standard); the longer the time, the worse the walking function^23^. The Wisconsin Gait Scale (WGS) was used to assess the gait performance of PwS. It has a total score of 42, with higher scores indicating poorer gait performance and more significant gait deviation^38^.

### Statistical analysis

All statistical analyses were conducted using IBM SPSS Statistics (Version 26.0) with α-level set at 0.05 for significance determinations. Between group comparisons of baseline characteristics including gender, type of stroke and lesion side were analyzed using Pearson’s chi-square test. Time to stroke and age violating normality assumptions were analyzed using Mann-Whitney U test. Temporal effects of gait parameters and clinical scales across four assessment points (baseline, 2-week, 4-week, 8-week) were examined using 2 × 4 mixed-design ANOVA: Between-subjects factor: intervention group (SBTT vs. TBTT) and Within-subject factor: Time. Mauchly’s test of sphericity was used to test covariance matrix sphericity. If the spherical assumption was not satisfied, the Greenhouse-Geisser method was used to adjust the degree of freedom to reduce the probability of a type I error. Significant interactions underwent post hoc pairwise comparisons with Bonferroni multiplicity correction. Bivariate relationships between clinical scale improvements (e.g. ΔLEFM, ΔBBS) and gait parameter changes (e.g. ΔSL, ΔSA, ΔTA) were quantified through Pearson correlation coefficients (r).

## Data Availability

The data is restricted by the Tianjin Union Medical Centre, in order to protect patients’ privacy. Data is available from corresponding author of the paper for researchers who meet the criteria for access to the confidential data.
